# Can Virtual Scientific Conferences Facilitate Two-Way Learning between Dementia Researchers and Participants?

**DOI:** 10.14283/jpad.2021.24

**Published:** 2021-05-25

**Authors:** Sarah Walter, B. Wheaton, C. Huling Hummel, J. Tyrone, J. Ziolkowski, E. Shaffer, N.T. Aggarwal

**Affiliations:** 1 Alzheimer's Therapeutic Research Institute, University of Southern California, 9860 Mesa Rim Road, 92121, San Diego, CA, USA; 2 Alzheimer's Clinical Trials Consortium Research Participant Advisory Board, Boston, USA; 3 Advisory Group on Risk Evidence Education for Dementia Stakeholder Sub-Committee, Miami, USA; 4 University of Michigan, Dearborn, USA; 5 Department of Neurological Sciences, Rush University Medical Center, Chicago, USA; 6 Rush Alzheimer's Disease Center, Rush University Medical Center, Chicago, USA

*Dear Editor*,

**T**he call to consider the perspective of research participants and care partners—referred to here as “participants”—in the design of studies on Alzheimer's disease (AD) and in dissemination of results has been increasing. Two-way learning between researchers and participants not only can potentially provide the information sought by participants, but it can also educate researchers on the lived experiences in dementia, thereby improving the quality of research and the applicability of outcomes (1, 2, 3). The inclusion of patients in medical conferences in other therapeutic areas, notably in AIDS, has had positive outcomes for both researchers and participants (4, 5); however, this practice has low acceptance in dementia research.

Responding to requests, we piloted a program to evaluate the feasibility of virtual scientific conferences that promote two-way learning. The abovementioned factors, along with the offering of the Alzheimer's Association International Conference 2020 in a free-of-charge digital format due to the COVID-19 pandemic, formed the backbone of this pilot, which aimed to determine the feasibility of inclusive scientific meetings and establish best practices. Specifically, we intended to evaluate (1) the interest in attendance, (2) what topics are of most interest, (3) how best to support participants' experiences, and (4) whether feedback on research design and approach could be gleaned from small-group discussions (SGDs).

Participants were invited from the Advisory Group on Risk Evidence Education for Dementia: Stakeholder Subcommittee and from the Alzheimer's Clinical Trials Consortium Research Participant Advisory Board. We employed targeted recruitment because (1) we had insufficient experience on virtual conferences and we needed to provide adequate technical support to our participants and (2) we had insufficient time to develop a comprehensive plan for outreach and recruitment. We provided support through an introductory webinar, through daily emails with highlighted sessions and navigation tips, and through a glossary of frequently used terms and acronyms.

Fourteen of the 20 invited participants registered for the conference, which lasted five days; most participants attended for at least two days and participated in SGDs. All participants had college education, and 60% were women; two were from underrepresented communities. Eight participants had a family history of AD or dementia, four had been diagnosed with mild AD or dementia, and six were current or former care partners.

In the pre-conference survey, the participants provided broad topics of interest, including clinical trials, genetic risks, dementia prevention, brain imaging, blood-based biomarkers, underrepresented populations, and caregiving. Other topics of interest included Tau, APOE, end-of-life preferences, domestic violence, correlation between AD and COVID-19, younger onset, participant support within and outside of clinical trials, and computer technology.

During SGDs, the participants who attended the same session highlighted varied takeaways, such as the realization on the impact of different healthcare systems on research results and the potential of digital tools in increasing accessibility and participation. The conference enhanced their understanding of clinical progress, confirmed their own experiences, and validated their current treatment strategy. The SGD on research findings expanded quickly into discussions on the potential impact of learnings on clinical care or research and on new research questions and approaches. For example, the participants recommended that researchers must address concerns about privacy upfront and must facilitate human contact, which participants highlighted as a key motivator for remaining in research studies.

This pilot illustrates that scientific conferences offer a unique opportunity for participants to interact with scientists and learn firsthand the breadth and depth of efforts underway to develop better therapies. Virtual conferences allow for the engagement and participation of individuals with limited mobility and time. A key strength of virtual conferences was highlighted by participants, that is, they can pause, rewind, search definitions of unfamiliar terms, as well as listen to a session more than once to better absorb information. They found the conference encouraging, fun, inspiring, and informative. The SGDs effectively supported their experiences and facilitated two-way learning. Setting expectations up front, being transparent about the desired outcomes, and providing support through emails and daily discussions were key to our success. Future work will compare the feedback and conference experiences of participants diagnosed with cognitive conditions and those without symptoms. We also plan to evaluate whether our strategies could effectively facilitate two-way learning in a more diverse group and potentially build trust in research, particularly among research-naïve individuals.

The association's decision to hold a free virtual conference was recognized as highly valuable, where attending in-person conferences would be expensive and burdensome. We urge researchers and conference organizers to include research participants and care partners in the development and conduct of future scientific conferences. We also hope that organizers will recognize the advantages of a virtual platform and will continue offering this engagement option free of charge, even when in-person conferences again become the norm.

*Funding:* This pilot was supported by the Alzheimer's Clinical Trials Consortium, which in turn is funded through a cooperative agreement by the National Institute on Aging, National Institutes of Health (U24AG057437).

*Conflict of Interest/Disclosure:* The authors received support through grants from the National Institute on Aging. None of the authors have additional financial interests, relationships, or affiliations relevant to the subject of this manuscript.

*Ethical Considerations:* No consent was required given that this work is not a human subject research per the assessment of our IRB.
